# Sleep‐wake variation in body temperature regulates tau phosphorylation, secretion and splicing

**DOI:** 10.1002/alz70855_107132

**Published:** 2025-12-25

**Authors:** Geoffrey Canet, Nicolas Sergeant, Brendan P Lucey, Esther M Blessing, Emmanuel Planel

**Affiliations:** ^1^ Université Laval ‐ Centre de Recherche du CHU de Québec, Québec, QC, Canada; ^2^ Univ. Lille, Inserm, CHU Lille, UMR‐S1172 ‐ Lille Neurosciences & Cognition, Lille, France; ^3^ Washington University School of Medicine, Saint Louis, MO, USA; ^4^ New York University, New York City, NY, USA

## Abstract

**Background:**

Aggregates of hyperphosphorylated tau protein are a hallmark of Alzheimer's disease (AD) and other tauopathies. Sleep disturbances—common in AD—have been associated with altered tau phosphorylation and secretion in both animal models and humans, and insufficient sleep is recognized as a potential risk factor for AD. Moreover, sleep deprivation affects mRNA splicing, polyadenylation, and miRNA‐mediated degradation. However, how tau phosphorylation, secretion, and splicing are physiologically regulated across the sleep–wake cycle remains unknown.

**Method:**

Here, we combine *in vitro, in vivo* and clinical methods to investigate whether tau phosphorylation, secretion and splicing are governed by circadian rhythms linked to sleep and body temperature (BT). To elucidate underlying mechanisms, we exposed neuronal cells to the physiological temperatures observed under each condition.

**Result:**

We found that tau phosphorylation undergoes sleep‐driven circadian variations, as it is hyperphosphorylated during sleep, when BT is lower. Similar changes in tau phosphorylation were reproduced in neuronal cells exposed to temperatures recorded during the sleep–wake cycle. In addition, we reveal a novel pathway by which BT modulates tau secretion, with higher temperature increasing extracellular and circulating tau levels. Tau splicing also varied with temperature, as lower temperatures favored exon 10 exclusion. Finally, in humans, the increase in CSF tau levels observed post‐wakefulness correlated with BT increase during wakefulness.

**Conclusion:**

Overall, these findings demonstrate that tau phosphorylation, secretion, and splicing follow a circadian pattern primarily regulated by BT and sleep. Given the prevalence of sleep disruptions and thermoregulatory deficits in AD, this study offers new insight into how tau pathology may develop and spread.

